# Cellular and Humoral Immunogenicity of a Candidate DNA Vaccine Expressing SARS-CoV-2 Spike Subunit 1

**DOI:** 10.3390/vaccines9080852

**Published:** 2021-08-04

**Authors:** Khalid A. Alluhaybi, Rahaf H. Alharbi, Rowa Y. Alhabbab, Najwa D. Aljehani, Sawsan S. Alamri, Mohammad Basabrain, Rehaf Alharbi, Wesam H. Abdulaal, Mohamed A. Alfaleh, Levi Tamming, Wanyue Zhang, Mazen Hassanain, Abdullah Algaissi, Adel M. Abuzenadah, Xuguang Li, Anwar M. Hashem

**Affiliations:** 1 Vaccines and Immunotherapy Unit, King Fahd Medical Research Center, King Abdulaziz University, Jeddah 21859, Saudi Arabia; Kalluhaybi@kau.edu.sa (K.A.A.); rahafh_h@live.com (R.H.A.); ralhabbab2@gmail.com (R.Y.A.); naljehani0053@stu.kau.edu.sa (N.D.A.); sawsan522@hotmail.com (S.S.A.); mohammad.basabrain@gmail.com (M.B.); rehaf-sa@hotmail.com (R.A.); maalfaleh@kau.edu.sa (M.A.A.); 2 Faculty of Pharmacy, King Abdulaziz University, Jeddah 21859, Saudi Arabia; 3 Department of Medical Laboratory Technology, Faculty of Applied Medical Sciences, King Abdulaziz University, Jeddah 21859, Saudi Arabia; aabuzenadah@kau.edu.sa; 4 Department of Biochemistry, Faculty of Science, King Abdulaziz University, Jeddah 21859, Saudi Arabia; whabdulaal@kau.edu.sa; 5 Centre for Biologics Evaluation, Biologics and Radiopharmaceutical Drug Directorate, Health Products and Food Branch (HPFB), Health Canada and WHO Collaborating Center for Standardization and Evaluation of Biologicals, Ottawa, ON K1A 0K9, Canada; ltamm055@uottawa.ca (L.T.); aliciazhang112@gmail.com (W.Z.); sean.li@canada.ca (X.L.); 6 Department of Biochemistry, Microbiology and Immunology, Faculty of Medicine, University of Ottawa, Ottawa, ON K1N 6N5, Canada; 7 Department of Surgery, Faculty of Medicine, King Saud University, Riyadh 11451, Saudi Arabia; mhassanain@ksu.edu.sa; 8 Department of Medical Laboratories Technology, College of Applied Medical Sciences, Jazan University, Jazan 45142, Saudi Arabia; aalgaissi@jazanu.edu.sa; 9 Medical Research Center, Jazan University, Jazan 45142, Saudi Arabia; 10 Department of Medical Microbiology and Parasitology, Faculty of Medicine, King Abdulaziz University, Jeddah 21859, Saudi Arabia

**Keywords:** SARS-CoV-2 vaccine, cellular and humoral immunogenicity, DNA vaccine

## Abstract

The urgent need for effective, safe and equitably accessible vaccines to tackle the ongoing spread of COVID-19 led researchers to generate vaccine candidates targeting varieties of immunogens of SARS-CoV-2. Because of its crucial role in mediating binding and entry to host cell and its proven safety profile, the subunit 1 (S1) of the spike protein represents an attractive immunogen for vaccine development. Here, we developed and assessed the immunogenicity of a DNA vaccine encoding the SARS-CoV-2 S1. Following in vitro confirmation and characterization, the humoral and cellular immune responses of our vaccine candidate (pVAX-S1) was evaluated in BALB/c mice using two different doses, 25 µg and 50 µg. Our data showed high levels of SARS-CoV-2 specific IgG and neutralizing antibodies in mice immunized with three doses of pVAX-S1. Analysis of the induced IgG subclasses showed a Th1-polarized immune response, as demonstrated by the significant elevation of spike-specific IgG2a and IgG2b, compared to IgG1. Furthermore, we found that the immunization of mice with three doses of 50 µg of pVAX-S1 could elicit significant memory CD4^+^ and CD8^+^ T cell responses. Taken together, our data indicate that pVAX-S1 is immunogenic and safe in mice and is worthy of further preclinical and clinical evaluation.

## 1. Introduction

The emergence and rapid spread of the severe acute respiratory syndrome-coronavirus-2 (SARS-CoV-2), the causative agent of the coronavirus disease 2019 (COVID-19) pandemic, represent a serious public health and economic burden to humanity [1,2,3,4]. While the majority of COVID-19 patients are either asymptomatic carriers or have mild symptoms, such as fever, myalgia and cough, millions have suffered from life-threatening acute respiratory infections and deaths. As of June 2021, around 178 million confirmed cases have been reported with at least 3.8 million deaths [5]. Furthermore, the high transmissibility rate of SARS-CoV-2 among humans as well as the emergence of new variants of concern (VOC) of the virus pose significant obstacles toward controlling its spread [6,7,8], highlighting the urgent need for the development of safe, effective and equitably accessible vaccines.

Hundreds of SARS-CoV-2 vaccines have been or are being developed using traditional and innovative technology platforms [9]. Several of these vaccines were approved for emergency use by multiple regulatory agencies across the globe. Examples of traditional vaccines include killed/inactivated vaccines which demonstrated safety and efficacy in humans despite the several potential risks that may still exist [10]. Other developers have adopted innovative technologies and/or novel approaches for antigen design, gene expression and vector optimization, including adenovirus-based vaccines [11,12,13,14] as well as mRNA vaccines [15]. Additional platforms being used include novel viral vectors, recombinant subunit proteins, nanoparticles and plasmid DNA [16,17,18].

The spike (S) protein of SARS-CoV-2 is composed of a globular head S1 subunit containing the receptor-binding domain (RBD), and a membrane-proximal S2 subunit containing the fusion machinery of the virus [19]. Most of these aforementioned vaccines rely on using either the full-length S protein or the RBD as the immunogen because of their critical roles in viral entry and host tropism [19,20], and ability to elicit protective immunity in animals and humans after vaccination or infection [9,10,21]. Use of full-length S protein as immunogen, however, could be associated with undesired responses by inducing non-neutralizing antibodies which may contribute to disease enhancement, immunopathological inflammation and fatality [22,23,24,25,26,27,28]. As such, targeting the S1 subunit could help minimize a potentially undesirable effect. Here, we evaluated the humoral and cellular immunogenicity of a plasmid DNA vaccine candidate expressing the S1 subunit of the S protein in an attempt to focus the immune response towards the neutralizing-epitope rich domains.

## 2. Materials and Methods

### 2.1. Generation of DNA Construct

SARS-CoV-2 S1 coding sequence was PCR amplified from codon-optimized full-length S gene (GenBank accession number: MN908947) synthesized by GenScript USA Inc. (Piscataway, NJ, USA). The coding sequence of S1 (1–681 aa) was subcloned into the mammalian expression vector pVAX1 under the control of the cytomegalovirus immediate-early promoter, denoted as pVAX-S1. The construct was cloned between *NheI* and *KpnI* restriction sites. The construct was then confirmed by restriction digestion and sequencing. Bulk endotoxin-free preparations of pVAX-S1 and empty vector (pVAX) were prepared using the GenElute™ HP Select Plasmid Gigaprep Kit (Sigma, Germany) for animal studies.

### 2.2. Detection of SARS-CoV-2 S1 Protein Expression by Western Blot

HEK-293A cells (70–90% confluent) in 6-well plates were transfected with 2 μg of pVAX-S1, pVAX1, or pcDNA3.1 expressing full-length S protein (pcDNA-S) using Lipofectamine 2000 Transfection Reagent (Invitrogen, Carlsbad, CA) according to the manufacturer’s instructions, followed by incubation at 37 °C in a 7% CO_2_ incubator for 48 h. After that, media were removed, and transfected cells were then washed with phosphate-buffered saline (PBS) and lysed with radioimmunoprecipitation assay buffer (RIPA buffer) (Sigma, Germany). The lysates were subjected to Western blot analysis to test protein expression using in-house anti-S (SARS-CoV-2) rabbit polyclonal antibodies at a 1:1000 dilution. These polyclonal antibodies were generated in house in rabbits using full-length SARS-CoV-2 recombinant S protein (Sino biological, Beijing, China) and found specific for SARS-CoV-2 S protein (Supplementary Figure S1). Additionally, β-actin was detected with anti β-actin antibodies at a 1:3000 dilution (OriGene Technologies, Inc., Rockville, MD, USA) as a loading control.

### 2.3. Detection of SARS-CoV-2 S1 Protein Expression by Immunofluorescence

HEK-293A cells were seeded on cell culture slide and incubated at 37 °C in a 7% CO_2_ incubator to be 70% confluent by the next day. Cells were then transfected with 1 μg of either pVAX-S1 or pVAX control plasmid using Lipofectamine 2000 Transfection Reagent (Invitrogen, Carlsbad, CA, USA) according to the manufacturer’s instructions and incubated at 37 °C in a 7% CO_2_ incubator for 36 h. The media was removed, cells were washed with PBS, fixed with 4% formaldehyde at 4 °C for 10 min and permeabilized with 0.2% PBS-Triton X-100 (PBS-Triton) at 4 °C for 20 min. Cells were washed twice with PBS-Triton and blocked with 2% goat serum/PBS-Triton at room temperature for 30 min. Cells were then stained with in-house rabbit anti-S primary polyclonal antibodies at a 1:1000 dilution in 2% goat serum/PBS-Triton at 4 °C for 1 h. This was followed by three washes and staining with Alexa Fluor-488 labeled goat anti-rabbit IgG H&L secondary antibody (Abcam, UK) at 1:500 dilution in blocking buffer in the dark at room temperature for 1 h. Cells were finally washed three times with PBS-Triton and mounted with VECTASHIELD with DAPI counterstain antifade mounting medium (Vector Laboratories, Inc., Burlingame, CA, USA). Images were captured using Olympus BX51 Fluorescence Microscope and analyzed using ImageJ 1.53e Software.

### 2.4. Immunization and Samples Collection

6- to 8-week-old female BALB/c mice were provided from King Fahd Medical Research Center (KFMRC) core animal facility, King Abdulaziz University (KAU). All animal experiments were done according to guidelines and the approval of the Animal Care and Use Committee (ACUC) at KFMRC and ethical approval from the bioethical committee at KAU (approval number 04-CEGMR-Bioeth-2020). Mice were randomly divided into three experimental groups (8 mice/group) and immunized with three doses of 25 μg or 50 μg of pVAX-S1, or 50 μg of pVAX on days 0, 21 and 42. Mice were immunized intramuscularly using customized needle-free Tropis system (PharmaJet, Golden, CO, USA). Serum samples were collected every three weeks and mice were euthanized on day 63 to collect final bleed and spleens for immune response analysis.

### 2.5. Binding Antibodies Measurement by Indirect ELISA

End-point titers of anti-S1 total IgG or its isotypes (IgG1, IgG2a and IgG2b) from immunized mice were determined by ELISA, as previously described [29]. Briefly, 96-well plates were coated with 1 μg/mL SARS-CoV-2 S1 protein (Sino Biological, Beijing, China) at 4 °C overnight. Plates were washed three times with PBS containing 0.1% Tween-20 (PBS-T) before blocking with 5% skim milk in PBS-T for 1 h at room temperature. After washing, 2-fold serial dilutions of mouse sera, starting from 1:100, were added to wells and incubated for 1 h at 37 °C. Then, peroxidase-conjugated rabbit anti-mouse IgG secondary antibodies (Sigma, Germany) were added at the recommended concentrations and incubated for 1 h at 37 °C. After extensive washing, 3,3′,5,5′-tetramethylbenzidine (TMB) substrate (KPL, Gaithersburg, MD, USA) was added for 30 min to develop a colorimetric reaction. Finally, the reaction was stopped with 0.16 M sulfuric acid, and absorbance was read spectrophotometrically at 450 nm on a Synergy 2 Multi-Detection Microplate Reader (BioTek, Winooski, VT, USA). End-point titers were determined as the reciprocals of the highest dilution with an OD above the cut-off value which was defined as the OD mean from the control group plus three standard deviations (SDs).

### 2.6. Neutralizing Antibodies Measurement by Pseudovirus Neutralization Assay

To assess the ability of induced antibodies to inhibit virus entry, pseudovirus neutralization assay was performed, as previously described [30]. Briefly, recombinant vesicular stomatitis virus, expressing codon-optimized full-length SARS-CoV-2 S protein (GenBank accession number: MN908947) (rVSV-ΔG/SARS-2-S*-luciferase pseudovirus), was generated in BHK21/WI-2 cells. Pseudovirus was collected and titrated on Vero E6 cells, as previously described [30]. Then, neutralization the assay was conducted by co-incubating two-fold serial dilutions of heat-inactivated mouse sera from vaccinated and control groups started from 1:20 dilution (in duplicate) with media containing 5 × 10^4^ relative luciferase unit (RLU) of rVSV-ΔG/SARS- 2-S*-luciferase pseudovirus for 1 h at 37 °C in a 7% CO_2_ incubator. The mixtures were then transferred onto confluent Vero E6 cell monolayers in white 96-well plates and incubated for 24 h at 37 °C in a 7% CO_2_ incubator. Following that, cells were lysed, luciferase activity was measured using Luciferase Assay System (Promega) according to the manufacturer’s instructions, and luminescence activity was measured using BioTek Synergy 2 microplate reader (BioTek, Winooski, VT, USA). Cell-only control (CC) and virus control (VC) were included with each assay run. The median inhibitory concentration (IC_50_) of neutralizing antibodies (nAbs) was determined using GraphPad Prism version 9.0.2 software.

### 2.7. Determination of T Cell Response by Flow Cytometry

Single cell suspensions of splenocytes were prepared from each mouse in immunized and control groups. One million splenocytes/well were re-stimulated with 5 μg/mL of a pool of 15-mer peptides overlapping with 11 amino acid and covering the SARS-CoV-2 S1 protein (GenScript USA Inc, Piscataway, NJ, USA) for 6 h at 37 °C in a 7% CO_2_ incubator in the presence of brefeldin A (BD Biosciences, San Jose, CA, USA) at a final concentration of 1:1000. Phorbol myristate acetate/ionomycin was used as a positive control, and RPMI 1640 medium was used as a negative unstimulated control. Cells were then washed in FACS buffer (PBS with 2% heat inactivated FBS) and stained with LIVE/DEAD™ Fixable Near-IR Dead Cell Stain Kit, for 633 or 635 nm excitation (Invitrogen, Carlsbad, CA, USA) for 30 min at room temperature. After washing with FACS buffer, cells were stained for surface markers with Pacific Blue-conjugated anti-mouse CD8, Pacific Blue-conjugated anti-mouse CD4, APC-conjugated anti-mouse CD44 antibody and Pe-Cy7-conjugated anti-mouse CD62L antibodies (BioLegend, UK). The cells were then washed with FACS buffer and fixed and permeabilized using Cytofix/Cytoperm Solution (BD Biosciences, San Jose, CA, USA) according to the manufacturer’s protocol. For intracellular staining, cells were labeled with FITC-conjugated anti–mouse IFN-γ (clone XMG1.2), PE-conjugated anti-mouse TNF-α (clone MP6-XT22) and PerCP/Cy5.5–conjugated anti-mouse IL-2 (clone JES6-5H4) antibodies (BioLegend, UK) for 20 min at 4 °C. Cells were then washed twice with permeabilization buffer and once with FACS buffer. All data were collected using BD FACSAria™ III flow cytometer (BD Biosciences, San Jose, CA, USA) and analyzed using FlowJo v10 software (Tree Star). Analysis of polyfunctional T cells was done by Boolean gating using FlowJo software from vaccinated animals, as previously described [31,32,33].

### 2.8. Statistical Analysis

Statistical analysis and graphical presentations were generated using GraphPad Prism version 9.0.2 software (Graph-Pad Software, Inc., CA, USA). Statistical analysis was conducted using the Mann–Whitney test or one-way analysis of variance with Bonferroni post hoc test to adjust for multiple comparisons between groups. All values are represented as mean ± SD and statistical significance is reported as *, *p* ≤ 0.05, **, *p* ≤ 0.01, ***, *p* ≤ 0.001, and ****, *p* ≤ 0.0001.

## 3. Results

### 3.1. In Vitro Confirmation of Protein Expression from the Candidate Vaccine

**Figure 1:**
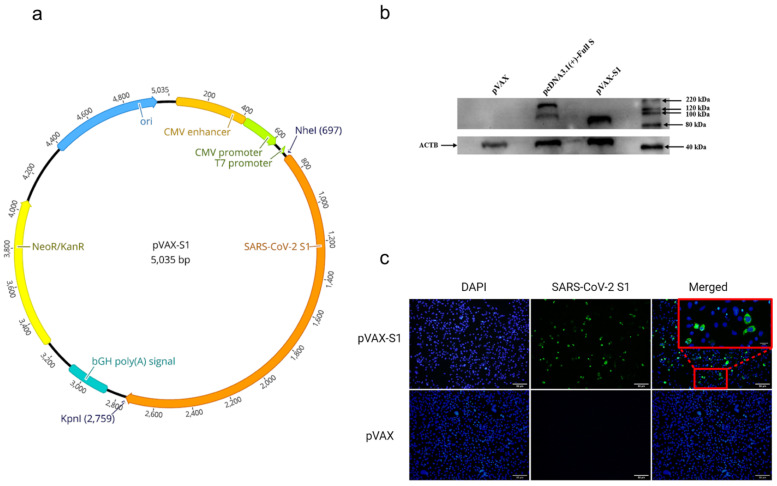
Vaccine design and characterization. (**a**) DNA vaccine (pVAX-S1) map. The inserted gene (SARS-CoV-2 S subunit 1) is indicated by orange color in the pVAX1 plasmid. (**b**) Western blot analysis. Figure shows bands of expressed full-length S from cells transfected with pcDNA3.1-Full S (positive control) and S1 subunit protein expressed from pVAX-S1. Empty pVAX was used as a negative control. (**c**) Immunofluorescence analysis. Cells transfected with pVAX-S1 or empty control pVAX were stained with anti-SARS-CoV-2 S rabbit polyclonal antibodies. Scale bars are 50 µm. Red square is magnified to scale bar of 10 µm. Merging and magnification were processed by ImageJ 1.53e.

The generated DNA vaccine candidate (Figure 1a) was evaluated for protein expression in vitro in HEK-293A cells prior to animal experiments. As shown in Figure 1b and Supplementary Figure S2, Western blot analysis confirmed that the recombinant construct was able to express S1 subunit protein at the expected molecular weight. A plasmid expressing full-length S protein (pcDNA3.1-Full S) was used as a positive control. Similarly, immunofluorescence analysis showed the expression of SARS-CoV-2 S1 protein in transfected cells (Figure 1c), suggesting that the expressed protein maintained structural confirmation to be detected by polyclonal anti-S antibodies. As expected, no protein was detected from cells transfected with the empty control plasmid pVAX (Figure 1b,c).

### 3.2. Evaluation of Binding and Neutralizing Antibodies in Immunized Mice

**Figure 2:**
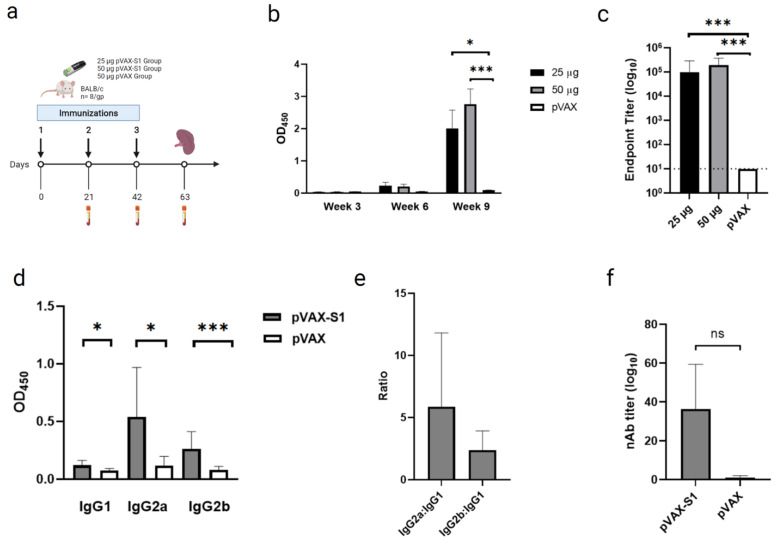
Humoral immunity in pVAX-S1 immunized mice. (**a**) Animal experimental plan. Mice were divided into three experimental groups (*n* = 8) and immunized intramuscularly with three doses on days 0, 21 and 42 using 25 μg or 50 μg of pVAX-S1, or 50 μg of pVAX1 using a customized needle-free Tropis system (PharmaJet, Golden, CO, USA). (**b**) Mean optical density (OD) values of S1-specific binding total IgG at 1:100 dilution as determined by ELISA at 3, 6 and 9 weeks after immunizations. (**c**) Mean end-point titers of S1-specific total IgG as determined by ELISA in samples collected on week 9. (**d**) Mean OD values of S1-specific IgG1, IgG2a and IgG2b in pVAX-S1 (50 μg) and pVAX immunized animals as determined by ELISA on week 9. (**e**) IgG2a:IgG1 and IgG2b:IgG1 ratios calculated from samples collected on week 9 from immunized mice in the group that received the 50 µg dose of pVAX-S1. (**f**) The median inhibitory concentration (IC_50_) of neutralizing antibodies (nAbs) was determined against rVSV-ΔG/SARS-2-S*-luciferase pseudovirus as described in the materials and methods. Data are depicted as mean ± SD. Statistical significance is reported as *, *p* ≤ 0.05, ***, *p* ≤ 0.001, and ns, not significant.

Mice were intramuscularly immunized with 3 doses of 25 μg or 50 μg of pVAX-S1 in a three-week interval regimen (Figure 2a). As a control, a group of mice was immunized with 50 μg of empty control vector (pVAX). Vaccine-induced binding antibodies were assessed by indirect ELISA from serum samples collected on weeks 3, 6 and 9. As shown in Figure 2b, analysis of S1-specific total IgG showed significant levels after only 3 doses with both doses of pVAX-S1 vaccine compared to control group (pVAX). No significant difference was found between 25 μg and 50 μg of pVAX-S1. Determination of end-point titers of S1-specific total IgG also confirmed the induction of significant levels of binding antibodies in samples collected on week 9 from each immunized mouse (Figure 2c). Testing the levels of IgG subclasses in samples collected on week 9 from mice immunized with 50 µg showed significantly higher levels of S1-specific IgG1, IgG2a and IgG2b compared to the pVAX control group (Figure 2d). Furthermore, the high IgG2a:IgG1 and IgG2b:IgG1 ratios suggested a Th1-skewed immune response, as shown in Figure 2e. To investigate whether the vaccine-induced antibodies were able to inhibit viral entry in cells, levels of nAbs were determined using pseudovirus neutralization assay from samples collected on week 9 in the 50 µg group. As shown in Figure 2f, immunized mice were only able to induce low levels of nAbs against SARS-CoV-2 pseudovirus in Vero cells. Collectively, these results confirm the ability of the vaccine to elicit significant Th1-skwed humoral immunity against SARS-CoV-2.

### 3.3. Evaluation of Cellular Immune Response in Immunized Mice

**Figure 3:**
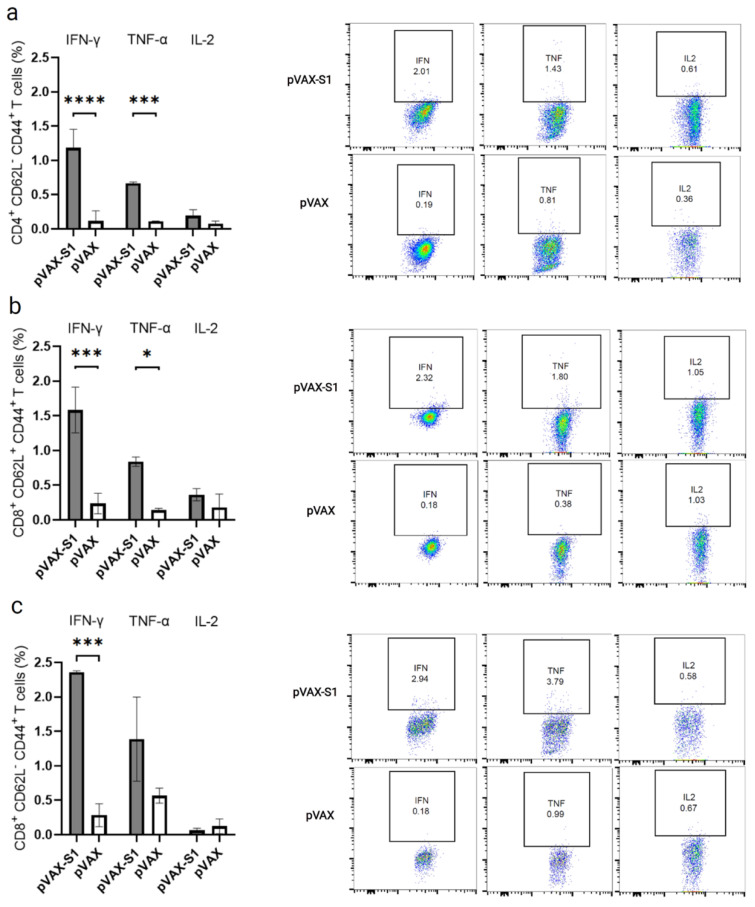
Overall memory T cells response in immunized mice. Histograms and FACS plots display overall IFN-γ, TNF-α and IL-2 expression from ex vivo re-stimulated (**a**) memory CD4 (CD4^+^CD62L^−^CD44^+^ T cells), (**b**) central memory CD8 (CD8^+^CD62L^+^CD44^+^ T cells) and (**c**) effector memory CD8 (CD8^+^CD62L^−^CD44^+^ T cells). Data in histograms are shown as percentages of induced cytokines from peptides-stimulated cells after subtracting levels produced by unstimulated splenocytes from each mouse. Representative FACS plots are shown. Data are shown as mean ± SD for each group from one experiment (*n* = 3). Statistical significance is reported as *, *p* ≤ 0.05, ***, *p* ≤ 0.001, and ****, *p* ≤ 0.0001.

Next, we investigated the overall memory CD4^+^ and CD8^+^ T cell response in 9-week samples (3 weeks post last immunization) collected from mice immunized with 50 μg dose of pVAX-S1 and pVAX. Re-stimulation with peptide pool covering the entire S1 protein resulted in significant levels of IFN-γ and TNF-α but not IL-2 from memory CD4^+^ T cells (CD4^+^CD62L^−^CD44^+^ T cells) from mice in pVAX-S1 group compered to control group (Figure 3a). Similarly, antigen-specific CD8^+^CD62L^+^CD44^+^ central memory T cells showed significant levels of IFN-γ and TNF-α but not IL-2 compared to pVAX control group (Figure 3b). On the other hand, effector CD8^+^CD62L^−^CD44^+^ memory T cells only produced IFN-γ at significant level compared to control group (Figure 3c).

**Figure 4:**
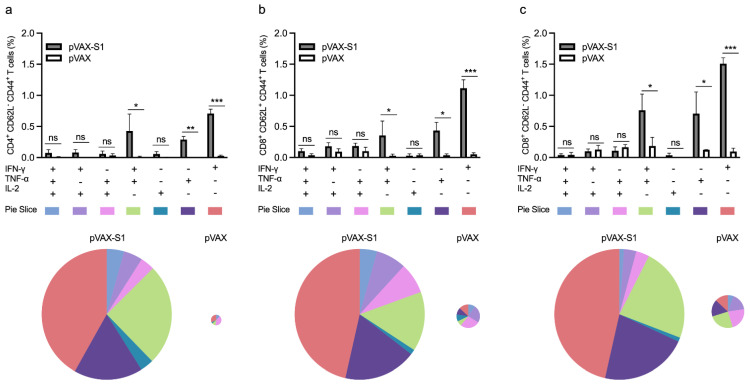
Polyfunctional memory T cell response in immunized mice. Bar graphs represent percentage of single-, double- and triple-cytokine–producing (**a**) memory CD4 (CD4^+^CD62L^−^CD44^+^ T cells), (**b**) central memory CD8 (CD8^+^CD62L^+^CD44^+^ T cells) and (**c**) effector memory CD8 (CD8^+^CD62L^−^CD44^+^ T cells) from samples collected on week 9. Data in histograms are shown as percentages of induced cytokines from peptide-stimulated cells after subtracting levels produced by unstimulated splenocytes from each mouse. Pie charts summarize the various combinations of cytokine-producing cells in each immunization group. Slices are grouped and color coded based on the number of functions. They represent the proportion of each single-, double- and triple-cytokine-producing cell in each subpopulation of (**a**) memory CD4 (CD4^+^CD62L^−^CD44^+^ T cells), (**b**) central memory CD8 (CD8^+^CD62L^+^CD44^+^ T cells) and (**c**) effector memory CD8 (CD8^+^CD62L^−^CD44^+^ T cells) T cells. The size of the pie chart represents the magnitude of the specific influenza virus immune response induced. Data are shown as mean ± SD for each group from one experiment (*n* = 3). Statistical significance is reported as *, **, *p* ≤ 0.01, *p* ≤ 0.05, ***, *p* ≤ 0.001, and ns, not significant.

Then, we looked at the polyfunctional (double- and triple-positive) as well as single-cytokine–producing subpopulations of memory CD4^+^ and CD8^+^ T cells from immunized mice as they represent a better indicator of the quality of cell-mediated immune response. Consistent with the overall secretion of S1-specific cytokines observed in Figure 3, cells producing IFN-γ and TNF-α as only cytokines were significantly higher in the pAVX-S1 group compared to pVAX (Figure 4). This was observed in all tested subpopulations of memory CD4^+^ and CD8^+^ T cells, despite the insignificant overall levels of TNF-α seen in effector CD8^+^CD62L^-^CD44^+^ memory T cells (Figure 3c). While we found some higher levels of S1-specific double- and triple-cytokines producing memory CD8^+^ and CD4^+^ T cells in pAVX-S1 immunized mice compared to the pVAX control group, only double-positive cells for both IFN-γ and TNF-α were found to be significantly higher in the pAVX-S1 group compared to pVAX immunized animals. Pie charts also show that single- (i.e., IFN-γ and TNF-α) and double-cytokine-producing cells (secreting both IFN-γ and TNF-α) dominated the immune response in immunized animals. Additionally, as shown by the size of the pie charts (Figure 4), pVAX-S1 elicited a greater overall magnitude of T cell responses, as compared to the pVAX group. Specifically, the magnitude of memory CD4 T cells, central memory CD8 T cells and effector memory CD8 T cells responses was higher by 20, 6.3 and 4.4 fold in pVAX-S1 group compared to pVAX group. Collectively, these data show that pVAX-S1 could elicit significant memory CD4^+^ and CD8^+^ T cell responses in mice.

## 4. Discussion

There is still an urgent need for multiple safe and protective vaccines against SARS-CoV-2 to combat the ongoing COVID-19 pandemic [9]. DNA-based vaccines represent a fast and safe approach to develop vaccines for such unprecedented situations [34]. Numerous studies on SARS-CoV-2 and other pathogenic human CoVs such as MERS-CoV and SARS-CoV have dementated that most of the nAbs that are generated due to either natural infection or full-length S based vaccines target the S1 subunit, making S1 an attractive and probably safer immunogen for vaccine development [35,36,37,38,39]. This is due to the fact that S1 contains the RBD and the N-terminal motif (NTD) which are critical for mediating binding to the host receptor. In this work, we successfully developed and evaluated the immunogenicity of a new DNA vaccine candidate against SARS-CoV-2, encoding the S1 subunit of the S protein. After the in vitro confirmation and characterization of S1 expression, we evaluated the immunogenicity and safety of the vaccine in mice. Overall, our data showed that pVAX-S1 was able to induce strong antibody responses in mice after a three-dose regimen of intramuscular immunization in a dose-dependent manner. Furthermore, we showed that pVAX-S1 induced a Th1-biased protective immune response, characterized by antibody production, predominantly, of IgG2a and IgG2b subclasses and the secretion of significantly elevated levels of Th1 cytokines (IFN-γ and TNF-α) produced by single- and double-cytokine-producing memory CD4^+^ and CD8^+^ T cells. Although the determination of Th2 cytokines levels from S1-specific T cells could have provided further evidence of the Th1 bias, the evaluation of IgG subclasses has been used as a surrogate to reflect such changes. Interestingly, while the vaccine candidate induced high levels of S1 specific Abs titers, we noticed that the level of the produced nAbs was relatively low, which should be investigated for further improvement. This may be through novel antigen design or the use of molecular adjuvants. Nonetheless, similar immune response induced by a similar DNA vaccine provided protection in non-human primates [40], suggesting protection through multiple mechanisms.

It is of note that several vaccine candidates have been developed using the S1 subunit as an immunogen, such as the DNA vaccine-expressing S1 domain with a foldon trimerization motif [40], live-attenuated YF17D, expressing S1 (YF-S1) [41], S1-Fc fusion subunit protein [42], and S1 subunit protein alone (S1) or fused to the norovirus shell domain (S1-S) [36]. These candidates used different technologies to test S1 immunogenicity in a number of animal models. Similar to our work, a single-dose of YF-S1 in hamsters or two doses of DNA vaccine-expressing S1-foldon in rhesus macaques induced significant levels of binding antibodies and low-to-medium levels of nAbs compared to other tested vaccines, expressing full-length cleavable S protein, prefusion-stabilized S or other truncated versions, such as those lacking the transmembrane or the cytoplasmic domains [40,41]. Additionally, consistent with our data, both of these S1-based vaccines induced highly elevated levels of Th1-skewed T cell responses compared to other vaccines [40,41]. Interestingly, while YF-S1 failed to protect hamsters from viral replication [41], S1-foldon DNA vaccine led to reduction in the viral RNA after SARS-CoV-2 challenge in rhesus macaques [40]. On the other hand, other developed subunit vaccines, such as S1-Fc and S1-S fusion proteins, elicited significantly high levels of nAbs in multiple animal models that exceeded the levels observed in acutely infected individuals [33,39]. These previous reports, as well as our current data, clearly show the potential of SARS-CoV-2 S1 as a promising immunogen [36,40,41,42].

The use of S1 as an immunogen has been proposed for other highly pathogenic coronaviruses, such as MERS-CoV and SARS-CoV, because of its potential high safety profile compared to the use of full-length S. Although full-length S protein can induce the highest immune response, some reports suggested its association with possible side effects in the currently used COVID-19 vaccines [22,23]. Additionally, previous reports on MERS-CoV, SARS-CoV and other coronaviruses have suggested that the use of a full-length S based vaccine could lead to undesired immune response upon infection [24,25,26,27,28]. Although the exact mechanism of this vaccination-induced immunopathology and/or disease enhancement has not been fully elucidated, it has been postulated that the non-neutralizing epitopes within the S protein may be responsible for the harmful immune response in vaccinated hosts [43,44]. These data suggested that using the neutralizing-epitope rich S1 subunit of the S protein could be a better approach to avoid any potential safety concerns.

Within the past year and half, several COVID-19 vaccines have been approved for emergency use with hundreds of others currently in different stages of clinical development [9,45,46]. Among those that have been approved or in late stages of clinical development are the nucleic acid-based vaccines. DNA and mRNA vaccines have several advantages over other platforms. For example, these vaccines can be developed rapidly without the need for the cultivation of the target pathogen and can be easily produced on a large industrial scale. Furthermore, nucleic acid-based vaccines can be rapidly and easily adapted to respond to potential mutations/variants. Compared to mRNA vaccines, which require a cold-chain system, DNA vaccines are more thermo-stable with less stringent storage conditions and easier formulation, enabling such technology to be a promising platform for wide distribution across the globe. While no major side effects associated with plasmid DNA vaccines were reported, especially for those developed for SARS-CoV-2 [18], reports on the generation of autoantibodies or anti-DNA antibodies are still controversial [47] and should be addressed in future studies, especially when high or multiple doses are used.

In conclusion, our approach using plasmid DNA (pVAX) to encode the SARS-CoV-2 S1 as a proof of principle has demonstrated that S1 can lead to high humoral and cellular immunity in mice with a predominant Th1-biased response. Such an approach can be further enhanced by the use of an efficient adjuvant and improved method of delivery.

## Data Availability

The raw data collected in this study are available on request from the corresponding author.

## Supplementary Materials

The following are available online at https://www.mdpi.com/article/10.3390/vaccines9080852/s1, Figure S1. Specificity of the in-house rabbit anti-S (SARS-CoV-2) polyclonal antibodies, Figure S2. Western blot analysis.
