# Supplementary material for: Cellular and Humoral Immunogenicity of a Candidate DNA Vaccine Expressing SARS-CoV-2 Spike Subunit 1
Source: Vaccines (Basel). 2021 Aug 4;9(8):852. doi: 10.3390/vaccines9080852 (PMC8402341; doi:10.3390/vaccines9080852)
Supplement: Supplementary file 1 [file vaccines-09-00852-s001.zip › vaccines-1294888-supplementary.pdf]

Supplementary Materials

Cellular and humoral immunogenicity of a candidate DNA vaccine expressing SARS-CoV-2 spike subunit 1

Khalid A Alluhaybi[1,2], Rahaf H Alharbi[1], Rowa Y Alhabbab[1,3], Najwa D Aljehani[1,4], Sawsan S Alamri[1,4], Mohammad Basabrain[1], Rehaf Alharbi[1], Wesam Abdulaal[4], Mohamed A Alfaleh[1,2], Levi Tamming[5,6], Wanyue Zhang[5,6], Mazen Hassanain[7], Abdullah Algaissi[8,9], Adel M. Abuzenadah[3], Xuguang Li[5,6], Anwar M Hashem[1,10*]

[1] Vaccines and Immunotherapy Unit, King Fahd Medical Research Center, King Abdulaziz University, Jeddah, Saudi Arabia
[2] Faculty of Pharmacy, King Abdulaziz University, Jeddah, Saudi Arabia
[3] Department of Medical Laboratory Technology, Faculty of Applied Medical Sciences, King Abdulaziz University, Jeddah, Saudi Arabia
[4] Department of Biochemistry, Faculty of Science, King Abdulaziz University, Jeddah, Saudi Arabia
[5] Centre for Biologics Evaluation, Biologics and Radiopharmaceutical Drug Directorate, Health Products and Food Branch (HPFB), Health Canada and WHO Collaborating Center for Standardization and Evaluation of Biologicals, Ottawa, ON, Canada
[6] Department of Biochemistry, Microbiology and Immunology, Faculty of Medicine, University of Ottawa, Ottawa, ON, Canada
[7] Department of Surgery, Faculty of Medicine, King Saud University, Riyadh, Saudi Arabia
[8] Department of Medical Laboratories Technology, College of Applied Medical Sciences, Jazan University, Jazan, Saudi Arabia
[9] Medical Research Center, Jazan University, Jazan, Saudi Arabia
[10] Department of Medical Microbiology and Parasitology, Faculty of Medicine, King Abdulaziz University, Jeddah, Saudi Arabia
*Correspondence: Anwar M Hashem; Faculty of Medicine and King Fahd Medical Research Center, King Abdulaziz University, P.O.Box 80205, Jeddah 21859, Saudi Arabia. Tel. +966 (12) 6400000 ext. 21033, E-mail: amhashem@kau.edu.sa

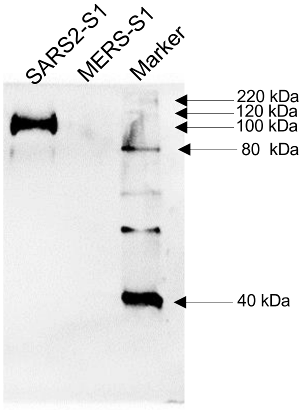

Figure S1. Specificity of the in-house rabbit anti-S (SARS-CoV-2) polyclonal antibodies. NZW rabbits were immunized subcutaneously with recombinant SARS-CoV-2 S protein (Sino biological, China) mixed with Freund's Complete Adjuvant at 100 µg per injection, and boosted twice with 100 µg per injection in Freund's Incomplete Adjuvant every two weeks. Serum was collected and Western blot was used to confirme specific binding of the polyclonal antibodies to S1 subunit proteins form SARS-CoV-2 but not MERS-CoV (Sino biological, China). All animal experiments were conducted in accordance with the Institutional Guidelines and Protocols for Animal Experiments.

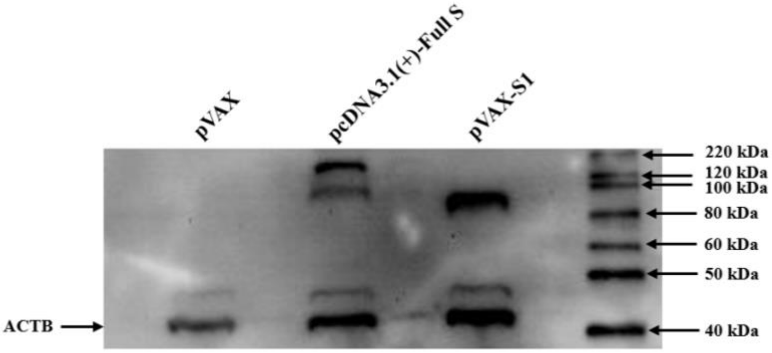

Figure S2. Western blots showing bands of expressed full-length S protein from cells transfected with pcDNA3.1-Full S (positive control) and S1 subunit protein expressed from pVAX-S1. Empty pVAX was used as negative control.
